# Evolution of Carotenoid Content, Antioxidant Activity and Volatiles Compounds in Dried Mango Fruits (*Mangifera Indica* L.)

**DOI:** 10.3390/foods9101424

**Published:** 2020-10-09

**Authors:** Alessandra Fratianni, Giuseppina Adiletta, Marisa Di Matteo, Gianfranco Panfili, Serena Niro, Carla Gentile, Vittorio Farina, Luciano Cinquanta, Onofrio Corona

**Affiliations:** 1 Dipartimento di Agricoltura, Ambiente e Alimenti, Università degli Studi del Molise, Via De Sanctis, 86100 Campobasso, Italy; fratianni@unimol.it (A.F.); panfili@unimol.it (G.P.); serena.niro@unimol.it (S.N.); 2 Dipartimento di Ingegneria Industriale, Università di Salerno, Via Giovanni Paolo II 132, 84084 Fisciano, Italy; mdimatteo@unisa.it; 3 Dipartimento di Scienze e Tecnologie Biologiche Chimiche e Farmaceutiche, Università di Palermo, Viale delle Scienze, Ed. 16, 90128 Palermo, Italy; carla.gentile@unipa.it; 4 Dipartimento Scienze Agrarie, Alimentari e Forestali, Università di Palermo, Viale delle Scienze 4, 90128 Palermo, Italy; vittorio.farina@unipa.it (V.F.); luciano.cinquanta@unipa.it (L.C.); onofrio.corona@unipa.it (O.C.)

**Keywords:** mango, drying, carotenoids, antioxidant activity, volatile compounds

## Abstract

The aim of this research was to study the evolution of carotenoid compounds, antioxidant β-ctivity, volatiles and sensory quality in two mango cultivars dried at 50, 60 and 70 °C. Total carotenoids in fresh samples were about 12 and 6 mg/100 g (dry basis) in Keitt and Osteen samples, respectively. β-carotene was the main carotenoid, representing about 50% of total carotenoids. In both cultivars, carotenoids were more susceptible to drying at 60 °C. Total phenols and metal reduction activity were higher in Osteen than in Keitt, which had higher values in radical scavenging capacity. The antioxidant activities were best preserved with drying temperatures at 50 °C in Keitt and 60 °C in Osteen fruits. Fresh Osteen mango fruits had a volatile compound content of about 37.1, while Keitt of about 5.2 mg/kg (dry basis). All the compounds with odorous impact were significantly reduced after drying. As regards organoleptic characteristics through sensory analysis, Keitt dried mangoes were quite similar to the fresh product, compared to Osteen.

## 1. Introduction

With a global production of about 40 million tons, mango (*Mangifera indica* L.) is one of the most important five tropical fruits, together with bananas, coconut, plantains and pineapple. Since the demand of the European market is growing, the cultivation of mangoes is increasing towards the Mediterranean areas. A niche production of mangoes has developed in Italy, on the Tyrrhenian coast of Sicily, where suitable conditions have been found for the production of fruits [1] rich in antioxidant phytochemicals such as polyphenols and carotenoids [2,3]. Furthermore, mango fruits have a pleasant taste and aroma, qualities that are fundamental for the sensory acceptance of consumers. The mango aroma consists mainly of terpenes, in addition to significant amounts of volatile oxygenated compounds, including furanones, esters and lactones, depending on the variety [4]. Nevertheless, being climacteric fruits, mangoes are highly perishable, due to their rapid ripening after harvesting, which causes deterioration of taste and texture, resulting in post-harvest losses, estimated at around 31% [5]. In order to reduce production losses due to quality detriment, the dehydration of sliced or diced fruits can be used, which reduces the water activity of the fruits, prolonging their shelf-life. The future market for dried mango will be determined by increased quality and availability during the year. The drying process is one of the most important methods to extend the fruit shelf-life, however, it may affect quality attributes. In particular, nutritional and health benefits can be strongly compromised due to the loss of unstable to heat nutrients and phytochemicals. Therefore, selecting and optimizing drying methods is important to preserve fruit quality. Several research works investigated the impact of drying processes on fruit antioxidant content, however, to date, there is a lack of studies which determine the phytochemical changes of dried mango fruits. With the aim to evaluate the stability of bioactive compounds during drying, in the present study, the carotenoid compounds, the polyphenolic content and the antioxidative properties of dried slices of mango fruits were assessed and compared with those of fresh fruits. These parameters were evaluated, together with volatile compounds and sensory qualities, on the fruit of two mango cultivars dehydrated at three different temperatures (50, 60 and 70 °C).

## 2. Materials and Methods

### 2.1. Plant Materials

Mango fruits (*Mangifera indica* L; cvs Keitt and Osteen) were harvested at the Cupitur Company in Acquedolci (Me), Sicily, Italy. Twenty-five fruits per cultivar (5 fruits × 5 trees × cv) were used as a sample and harvested by hand at commercial ripeness. For physico-chemical and sensory analysis fifteen fruits were used, while the other 10 fruits were dried.

### 2.2. Standards and Chemicals

[2,2′-azinobis(3-ethylbenzothiazoline-6-sulfonic acid)]-diammonium salt (ABTS), Folin–Ciocalteu’s reagent, 6-hydroxy-2, 5,7,8-tetramethylchroman-2-carboxylic acid (Trolox), gallic acid (GA), potassium chloride, potassium persulfate, 2,4, 6- tripyridyl-S-triazine (TPTZ) and iron (III) chloride hexahydrate (FeCl_3_ 6H_2_O) were purchased from Sigma-Aldrich. Ethanol (LC-MS grade) was purchased from Biosolve B.V. (Valkenswaard, The Netherlands). All other materials and solvents were of analytical grade.

### 2.3. Physico-Chemical Analyses

The moisture content of selected samples was determined by the oven-drying method at 105 °C [6]. To measure the water activity (aw) of dried fruits, a water activity meter (Testo 650, Testo Inc., West Chester, PA, USA) was used at 25 °C. Total soluble solids (TSS) were measured by a digital refractometer (°Brix) Atago Palette PR-32 (Atago Co., Ltd., Tokyo, Japan) and total acidity (g citric acid/L) using a CrisonS compact tritator (Crison Instruments, SA, Barcelona, Spain). Fresh samples were analyzed and dried within 24 h of harvest.

### 2.4. Drying Experiments

Prior to drying experiments, Keitt and Osteen mangoes were selected for the absence of defects and any mechanical damage, for freshness and uniformity of color and size. Afterwards, they were washed with tap water, peeled and cut into cylindrical slices with a diameter of 30 ± 0.25 mm and thickness of 5 ± 0.10 mm, randomly using several raw materials from each cultivar. Drying experiments were carried out in a conventional convective dryer (B80 FCV/E6L3, Termaks, Norway) at 2.1 m/s (air velocity), at 50, 60 and 70 °C. During the drying process, the mass of the sample was measured at regular intervals and all slices were dried to an average moisture of 0.30 ± 0.02 Kg water/kg dry weight and a water activity of about 0.45 ± 0.02, values that guarantee the microbiological stability of the fruit. At each temperature, the drying tests were conducted in triplicates.

### 2.5. Fruit Extract Preparation for Analyses

Dried samples (about 200 g) were finely chopped and then homogenized. One gram of the whole homogenate was extracted using ethanol with a ratio of 1:30 (*w/v*). Samples were vigorously mixed with a vortex for 5 min, sonicated for 10 min at room temperature and then incubated at the same temperature. After centrifugation (10 min at 8000× *g* at 4 °C), the supernatants were recovered, filtered and stored at −20 °C. In order to obtain three different technical replicates, the procedure of extraction was repeated another two times.

### 2.6. Total Phenolic Content and Total Flavonoid Content

The total hydrophilic reducing compounds were represented mainly by phenols. The total phenolic content (TPC) was determined by the Folin–Ciocalteu method [7]. An external calibration curve with gallic acid (GA) was used for quantification, and the results were expressed as mg GA equivalents (GAE) per g on a dried basis (d.b.). Four repeated measurements were made.

The total flavonoid content was determined with a colorimetric aluminum chloride method as described by Herald et al. [8].

### 2.7. Radical Scavenging and Reducing Activities in Solution

ABTS assay. The decolorization assay based on ABTS radical cation was performed as previously described [9]. The method monitors the decay of the colorization of ABTS^•+^, produced by the reaction of ABTS with potassium persulfate, recorded at 515 nm. Five different dilutions of samples were analyzed, within the linearity range of the assay. All experiments were repeated three times and the results were expressed as μmol Trolox Equivalent (TE) per g d.b. 

DPPH assay. The decay of the colorization of DPPH was monitored and recorded at 735 nm [10]. The experiments were repeated three times and the results were expressed as μmol TE per g of d.b. 

FRAP assay. Total antioxidant activity of fruit extracts was measured by ferric reducing antioxidant power (FRAP) assay, relying on the reduction of the Fe^3+^–TPTZ complex to the ferrous form [11]. Briefly, the FRAP reactive (0.3 M acetate buffer pH 3.6, 10 mM TPTZ and 20 mM FeCl_3_, (8:1:1, *v/v*)) was mixed with an appropriate sample dilution and incubated at 37 °C for 30 min; then, the absorbance was read at 595 nm. All measurements were repeated in triplicate and the results were expressed as μmol TE per g of d.b.

(1) $$CAA\;=\;100-\left[\frac{\int SA}{\int CA}\right]\times 100$$

### 2.8. Carotenoids Extraction and Quantification

The procedure for carotenoids extraction was the saponification method of Panfili et al., 2004 [12], involving the saponification of the food matrix. Compounds were analyzed through the combination of a normal- and a reverse-phase HPLC method. A HPLC Dionex (Sunnyvale, CA) analytical system, consisting of a 50 μL injector loop (Rheodyne, Cotati) and a U6000 pump system, was used. For the normal phase (NP), the mobile phase was n-hexane/isopropyl alcohol in multilinear gradient elution from 10% (A) to 20% (B) of isopropyl alcohol, with a flow rate of 1.5 mL/min. The other chromatographic conditions are reported elsewhere [12]. For the reverse phase (RP), the organic dry residues were suspended in methanol/MTBE 50:50 (*v/v*) and the original method, elsewhere reported [13], was applied. β-Cryptoxanthin was from Extrasynthese (Z.I. Lyon-Nord, Genay, France), all-trans-β- carotene was from Sigma Chemicals (St. Luis, MO, USA), all other standards were from CaroteNature (Lupsingen, Switzerland). Spectral characteristics and comparison of retention times with those of the available standard solutions were used to identify carotenoids. Total carotenoids were the sum of single quantified carotenoids. Samples were analyzed in triplicate.

### 2.9. Volatile Organic Compounds (VOCs)

The extracts of mango fruits were submitted to GC/MS analysis to detect the volatile organic compounds (VOCs), whose extractions were carried out using an SPME fiber of divinylbenzene/carboxen/polydimethylsiloxane (Supelco, Bellefonte, PA, USA). Conditioning of the fiber was done at a temperature of 250 °C for 30 min, which was then subjected to an exposure step for 30 min at 40 °C to the headspace of the sample vial. The GC/MS equipment, conditions, column used for analysis and the identification of different compounds were described elsewhere [14,15]. Samples were analyzed in duplicate.

### 2.10. Sensorial Analysis

Evaluation of the sensory profiles of the mango fruits was performed using a descriptive method as previously reported [16]. The only difference from the method is the number of selected sensory attributes, which here is six.

### 2.11. Statistical Analysis

Analysis of variance (ANOVA) and Tukey’s multiple range test or honestly significant difference (HSD) for *p* ≤ 0.05 were used to investigate the differences between all data. The differences between Keitt and Osteen cultivars were determined using the Student’s test. All statistical analyses were realized by SYSTAT 10 software (Systat Software, Inc., San Jose, CA, USA) 

## 3. Results and Discussion

### 3.1. Fresh Fruit Composition and Drying

**Figure 1:**
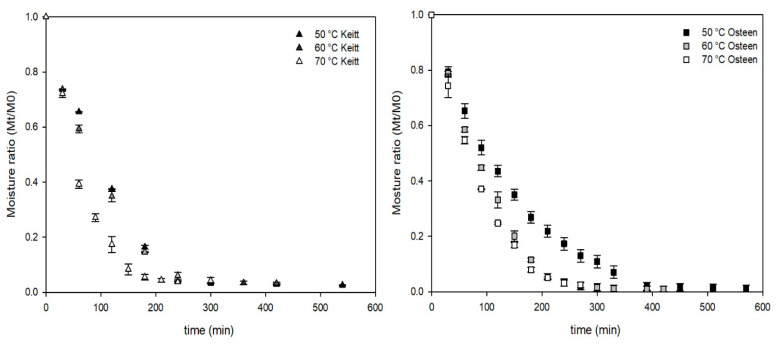
Drying curves of Keitt mango slices (**a**) and Osteen mango slices (**b**) at different temperatures (50, 60 and 70 °C). Moisture ratio (Mt/M0) is calculated as the ratio between the actual (Mt) and the initial (M0) moisture content on a dry basis.

The two mango fruits cultivars, although both are late-ripening, differ in their main chemical characteristics. The fresh mangoes had different total soluble solid contents (Keitt: 15.1; Osteen: 18.1 °Brix) and titratable acidity (Keitt: 0.31; Osteen: 0.10 g/L as citric acid). These values are comparable with those found in the literature [17] and compatible with immediate commercialization. The average value for moisture content in Keitt and Osteen fresh mangoes was 5.25  ±  0.18 (kg water/kg dry weight). The drying time to reach an average moisture of 0.30  ±  0.02 (kg water/kg dry weight) was about 270, 250 and 210 min for Keitt mango fruits, and 420, 300 and 260 min for Osteen ones, at 50, 60 and 70 °C, respectively (Figure 1a,b). Since the initial moisture content was the same for the two cultivars, this behavior is probably related to the different chemical composition and structure of the two cultivars. These data are in agreement with what has been reported elsewhere [18].

### 3.2. Total Phenolic Content and Antioxidative Properties 

**Figure 2:**
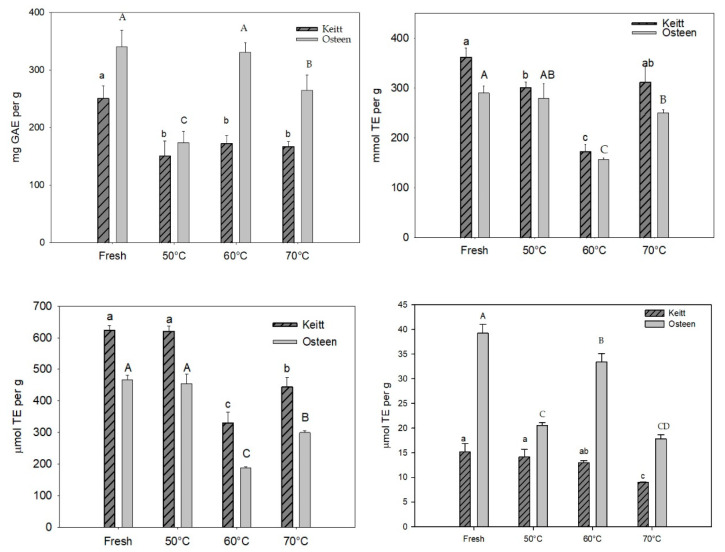
Total hydrophilic reducing compounds, expressed as gallic acid equivalent (GAE) (**a**); radical scavenging ability, evaluated as the ABTS value (**b**); radical scavenging ability, evaluated as the DPPH value (**c**); metal reducing activity, expressed as the ferric reducing antioxidant power (FRAP) value (**d**), in fresh and dried (50, 60 and 70 °C) Keitt and Osteen mango slices. TE = trolox equivalent.

TPC and antioxidative properties of the fresh pulp of mango fruits are reported in Figure 2. In the studied samples, TPC was higher in Osteen than in Keitt (Figure 2a), according to previously reported data [2]. Similarly, metal reducing activity, expressed as the FRAP value, was higher in Osteen than in Keitt (Figure 2d). On the contrary, concerning radical scavenging ability, as evaluated in both ABTS (Figure 2b) and DPPH (Figure 2c) assays, higher values were found in Keitt than in Osteen.

Metal reducing activity of polyphenolic compounds may also involve early metal complexation, through bidentate binding sites related to the presence of multiple OH groups and carbonyl moiety [19]. On the other hand, the ability to complex metal ions, involved in radical formation, is documented for a large number of polyphenols and contributes, with their radical scavenging properties, to the antioxidative activity of these phytochemicals [20]. The results suggest that the polyphenols in Osteen could have more pronounced metal chelating properties than radical scavenging properties in comparison with those in Keitt. The evolution of antioxidative properties of mango fruits during the drying process is shown in Figure 2. Concerning the Keitt genotype, the drying process reduced TPC by about 40%, regardless of the drying temperature (Figure 2a). On the contrary, concerning antioxidative properties, the DPPH value, which was stable when the drying temperature was 50 °C, decreased for higher temperatures.

A similar behavior was observed when radical scavenging activity was measured by ABTS assay: antioxidant properties were preserved at 50 °C, while they strongly decreased at 60 °C. Unexpectedly, the fall in the DPPH values was more limited at 70 °C than at 60 °C and ABTS values at 70 °C did not differ significantly from what was recorded for fresh fruit.

Concerning FRAP values of the Keitt genotype, in contrast with what was observed in the radical scavenging assays, only at 70 °C a decrease was recorded. These results suggest that components with metal chelating activities in mango fruits are more resistant to drying at medium temperatures than components with radical scavenging activity.

Concerning the Osteen genotype, the drying process reduced TPC both at 50 and 70 °C. On the contrary, TPC at 60 °C did not differ significantly from the recorded value for fresh fruit. The obtained results could be interpreted admitting that a too low temperature, delaying the dehydration of the sample, could promote oxidative reactions. A faster elimination of moisture, as it occurs at higher temperatures, could contain these degradation processes. A similar evolution to that observed for TPC was observed for the metal reducing activity, expressed as the FRAP value (Figure 2d). The correlation between the variations in TPC and the FRAP values during drying processes suggests that fruits from Osteen genotypes contain many polyphenols with metal chelating ability.

Total flavonoids represented on fresh fruits about 70% of TPC in the Keitt genotype and 80% in Osteen (data not reported). After the drying process, a similar evolution to that observed for TPC was detected for total flavonoids. Their loss was related to temperature and microstructure [21], for instance, Santhirasegaram et al. [22] reported a 25% reduction in total flavonoid content in mango juice after pasteurization at 90 °C for 6 s.

Evaluating the changes in the radical scavenging activity during drying processes, a similar behavior was observed for both cvs. In particular, antioxidant activity evaluated by ABTS and DPPH assays remained stable or slightly reduced at 50 °C, while drastically falling at 60 °C (Figure 2b,c). In this case, the unexpected increase in the ABTS and DPPH values from 60 to 70 °C could result from Maillard products formed at high temperatures.

In conclusion, the obtained results revealed that the temperature of drying processes affected functional properties of mango fruits with changes influenced by genetic factors due to the specific phytochemical profile of each cultivar. In particular, under this experimental condition, to preserve the antioxidative properties, it is preferable to process Keitt at 50 °C and Osteen at 60 °C.

### 3.3. Carotenoid Compounds

For carotenoid determination, reverse-phase (RP) C30 columns allow a better separation and a high resolution of geometric isomers of the less polar carotenes (mainly of β-carotene). However, the more polar xanthophylls are often co-eluted and are difficult to quantify. On the contrary, normal-phase HPLC is more efficient in separating polar xanthophylls (mainly the epoxycarotenoids) and their geometric isomers, which often co-elute in C30 columns. Since in the investigated mango samples we observed the presence of both polar xanthophylls and carotene isomers, in order to have a better quantification of the single compounds, a combination of methods involving NP- and RP-HPLC was used. By using RP-HPLC, the following main carotenoids were detected and quantified: β-criptoxanthin, 13-cis-β-carotene, β-carotene and 9-cis-β-carotene; through NP-HPLC, the epoxycarotenoids luteoxanthin, violaxanthin and neoxanthin were eluted and determined.

The epoxycarotenoids and isomers of β-carotene have been also detected by other authors in mango fruit [13]. β-carotene is the main detected compound, about 50% of total carotenoids, followed by β-criptoxanthin, luteoxanthin, violaxanthin and 9-cis-β-carotene (on average about 10%). Other compounds were present at lower amounts (about 5% or less).

Differences in carotenoid content were found between cultivars. In particular, total carotenoid amounts in fresh samples were about 12 and 6 mg/100 g d.b., in the Keitt and Osteen cultivars, respectively.

The found qualitative and quantitative composition is in accordance with what was reported by different literature data [2,23,24]. The profiles of total single carotenoids in fresh and dried samples at the end of each drying treatment, for both cultivars, are reported in Table 1. 

A general significant decrease in all compounds was found after drying, at all the used drying temperatures. In particular, in both cultivars, total carotenoids seemed more susceptible to the treatment at 60 °C (about 70% loss). About 40–55% (Keitt) and 50–35% (Osteen) losses were found at 50 and 70 °C, respectively. This behavior reflected that of β carotene, which, however, in the Osteen cultivar, at 70 °C, seemed not to be significantly affected by drying (*p* < 0.05). In processed mangoes, some of the most susceptible carotenoids to temperature were the epoxycarotenoids: luteoxanthin, violaxanthin and neoxanthin. In the Keitt cultivar, at 70 °C, a general decrease from about 50% for luteoxanthin and from about 70% for violaxanthin was observed. In the cultivar Osteen, a more marked loss of these compounds was found; in particular, with the increasing temperature, several small peaks, probably isomers, were detected in place of epoxycarotenoids. The severity and length of heat treatment can induce different carotenoid losses and isomerization [25]; moreover, these phenomena could also depend on the structure and cellular organization of carotenoids in the food matrix, oxygen occurrence, pH, water activity and the interactions with other antioxidants [26,27]. Previous works [28,29] and other authors [30] have shown the high susceptibility of epoxycarotenoids to thermal treatments and their isomerization into their epoxyderivatives. Different studies also report the concomitant decrease in all-trans-β-carotene and the rise in cis isomers during the thermal processing of vegetables, also in mango [26,31]; however, in our case, the degradation process seemed predominant upon isomerization in all cultivars, since losses (from 60% to 100%) were found, at all the tested drying temperatures.

Table 2 reports the values of vitamin A activity, expressed as retinol equivalent (R.E.), provided by 100 g of fresh weight (f.w.) and by a portion, which is 150 g for fresh mango and 30 g for dried mangoes [32]. Fresh mangoes provided an R.E. of 215 (Keitt) and 110 μg/100 g f.w. (Osteen). Taking into account the Recommended Daily Allowance (R.D.A) for vitamin A, which is 800 µg/day [33], 100 g of fresh mango contribute to 27% and 14% of the R.D.A, in Keitt and Osteen cultivars, respectively. In dried mangoes, the R.E. values increase as to fresh mangoes, with the lowest value in Osteen at 60 °C (about 210 µg/100 g f.w.), and the highest value in Keitt at 50 °C (about 650 µg/100 g/100 f.w.). Considering a single portion, with the exception of a portion of dried mangoes at 60 °C, all samples contribute more than 15% of the R.D.A., so as to be declared as a “source of vitamin A”.

### 3.4. PCA

**Figure 3:**
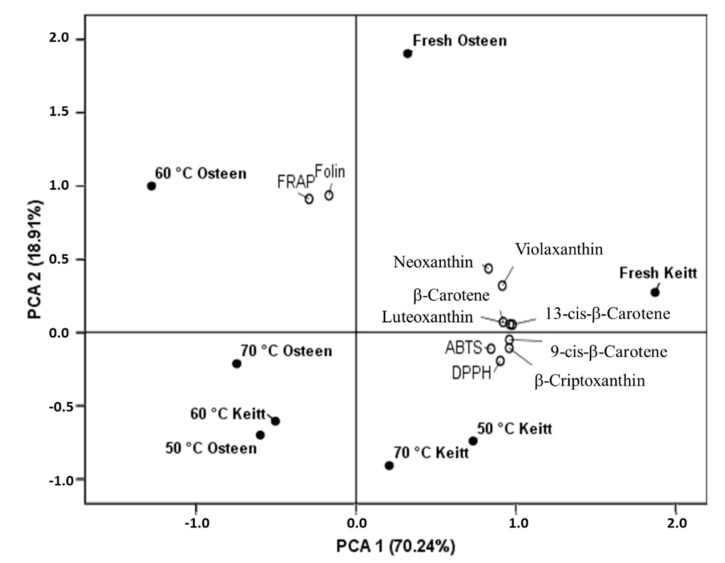
Principal component analysis of the carotenoids, total phenols (Folin), radical scavenging ability (ABTS and DPPH) and metal reducing activity (FRAP) in fresh and dried (50, 60 and 70 °C) Keitt and Osteen mango slices.

PCA 1 and PCA 2 together explained 89.15% of the total variability. The score plot (Figure 3) clearly shows the distance among the fresh and dried Keitt and Osteen mangoes. A strict relation was found between fresh Keitt mangoes and after drying at 50 and 70 °C, along positive PCA 1, while the highest differences were found for fresh and dried Osteen mangoes on opposite sides of PCA 1, which has the highest incidence (70.24%) on the total variability. Along PCA 2, which has a lower incidence (18.91%), a relevant difference was found for the fresh and dried mangoes. PCA 1 was mainly affected by all single carotenoid compounds, ABST and DPPH that showed the highest loading values, while the variability associated with PCA 2 was mainly explained by Folin and FRAP.

### 3.5. Volatile Compounds

#### 3.5.1. Volatile Compounds in Fresh Mangoes

A total of seventy-two volatile compounds (VOCs) were identified in fresh and dried mangoes. These included, among others, nine sesquiterpenes, ten monoterpenic hydrocarbons, fourteen acids and sixteen esters. Fruits of fresh Osteen mango had the highest VOCs: about 37.1 mg/kg d.b., compared to Keitt fruit with about 5.2 mg/kg d.b (Supplementary Table S1). Monoterpenes were found at the highest concentration in both analyzed cvs, 63% in Keitt and 90% in Osteen mangoes. The major VOCs in fresh Keitt mango fruits were α-pinene (2192 μg/kg d.b.) and α-fenchene (311 μg/kg d.b.), followed by γ-octalattone (257 μg/kg d.b.), hexadecanoic acid (217 μg/kg d.b.) and α-terpinene (188 μg/kg d.b.). In fresh Osteen, the VOCs concentrations were on average one order of magnitude higher than in the cv Keitt, the major VOCs were α-pinene (26015 μg/kg d.b.), α-fenchene (3207 μg/kg d.b.), α-terpinolen (1956 μg/kg d.b.), β-caryophyllene (1675 μg/kg d.b.) and D-limonene (1127 μg/kg d.b.). The concentration of α-pinene was reported to be 2.56 and 7.92 µL/L in mature green and tree-ripe mangoes, respectively [4]. As it is known, the contribution of the VOCs to the odor of the fruit depended not only on their quantities, but also mainly on their perception threshold. In fresh mango fruits, VOCs with an odor activity value (OAV) > 1 were: α-pinene, β-ionone, phenol, nonanal, ethyl 3-hydroxy butanoate, D-limonene, hexadecanoic acid, decanal, 1-dodecanol, β-terpinolene, α-fenchene and γ-terpinene (Table 3) [34,35,36,37,38]. These compounds, having fruity, woody, pine, grassy, phenolic, fatty, wax, citrus, green, floral, sweet, honey, green, berry, floral, citrusy, coniferous and camphoraceous odors, contributed to the characteristic aroma of mango. VOCs were also highlighted in other studies on mango [18,39,40,41].

#### 3.5.2. Volatile Compounds in Dried Mangoes

Noteworthy changes occurred in the volatile profile of mango fruit after drying (Supplementary Table S1): at 50 °C, the total amount of VOCs decreased more in Osteen mangoes, which started from significantly higher concentrations, than in Keitt fruits. The higher the drying temperatures, the lower the VOCs concentrations in Osteen mango, while in dried Keitt, there were no differences at 50 and 60 °C, with higher losses of VOCs at 70 °C. Further, in dried fruits, monoterpenic hydrocarbons were the main chemical class of VOCs, representing about 80% of total VOCs at 50 and 60 °C and about 52% at 7 °C. Among them, pinene and fenchene were the most abundant. Aldehydes, sesquiterpenes and esters were the prevalent compounds in the cv Keitt after drying, while acids, aldehydes and sesquiterpenes were prevalent in the cv Osteen.

Some aldehydes, such as hexanal, heptanal, trans-2-nonenal and 2-6-nonadienal, and some esters, such as ethyl butanoate, ethyl hexanoate and ethyl heptanoate, were detected only in dried mangoes. Monoterpenes, such as δ-3-carene, β-ocimene and isoterpinolene, and sequiterpenes, such as α-copaene, β-caryophyllen, α-caryophyllene, β-selinene, valencene and α-selinene, increased at 50 °C or were detected only in dried mangoes. These neoformation compounds could be generated through chemical and enzymatic reactions: aldehydes from fatty acids due to the effect of oxygen and heat. An increase in hexanal was found during the heating of paprika, in dried carrots and in peppers, due to auto-oxidative degradation of linoleic acid [42]. The increase in terpenes may result from hydrolysis of the glycosylate aroma precursor, while an increase in esters has been found in carob roasting [41], so some esters may be formed by heat treatment.

All the compounds with odorous impact of the mango, and not just them, were significantly reduced after drying. The compounds with OAV > 1 were always those observed in the fresh mango fruits (Table 3), with the addition of the trans-2-nonenal, ethyl butanoate, ethyl hexanoate and ethyl heptanoate, with apple and fruity odors. Lastly, several studies about mango showed that VOCs were highly dependent on the variety, the area of production, as well as the drying process [18,39,40,41].

### 3.6. Sensory Analysis

**Figure 4:**
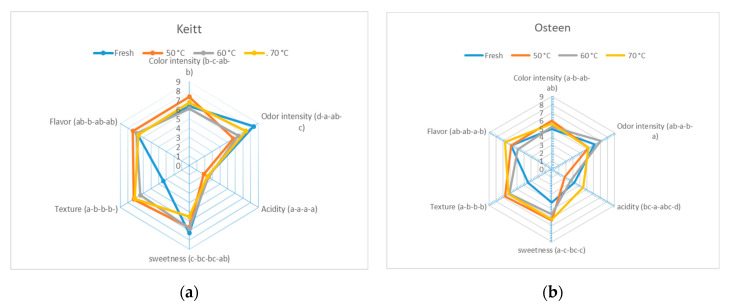
Sensory profile of the fresh and dried fruits of the cv Keitt (**a**) and the cv Osteen (**b**).

As regards organoleptic characteristics detected through sensory analysis, Keitt dried mangoes were quite similar to the fresh product, compared to Osteen. In dried Keitt, more significant variations in consistency and odor parameters were perceived [43] (Figure 4a). Keitt dried at 50 °C were considered to have better color and flavor intensity and a worse odor. In dried Osteen, the sweet character was particularly perceived (Figure 4b), due to its high concentration in the fresh fruit (18.1 °Brix). Osteen samples dried at 60 °C had significantly higher values for perceived odorous intensity, while acidity and flavor were higher in samples dried at 70 °C. Further, in Osteen, the color intensity was higher in the dried fruits at 50 °C than in the other samples. This result may be correlated with the higher carotenoid content found in mango samples dried at 50 °C (Table 1).

## 4. Conclusions

This research has studied the changes in the quality characteristics of two mango cultivars (Keitt and Osteen) grown in Sicily (Italy) and dried at 50, 60 and 70 °C. The results showed that the drying temperatures influenced the composition and functional properties of the mango fruits, according to the specific phytochemical profile of the two cvs. From the evaluation of antioxidant activities, volatile compounds and sensory analysis, the temperature of 50 °C better preserved the typical characteristics of the fresh fruits. On the other hand, drying at 50 °C required significantly longer drying times, up to almost 40% more than at 70 °C, in Osteen mangoes. β-carotene accounted for about the 50% of the total carotenoids, followed by β-criptoxanthin, luteoxanthin, violaxanthin and 9-cis-β-carotene (about 10%). As a result of the drying process, the most susceptible carotenoids were the epoxycarotenoids and in both cultivars the drying temperature at 60 °C caused a greater loss in carotenoids. Among the volatile substances, monoterpenes were found at the highest concentration in both fresh and dried fruits, where they decreased relatively slightly at lower temperatures. Concerning taste characteristics by sensory analysis, the dried Keitt mangoes were quite similar to the fresh product, with the exception of the texture parameter.

## Figures and Tables

**Table 1:** Content of the main carotenoids (mean ± standard deviation) in mango cultivars during thermal treatments at different temperatures (mg/100g d.w.).

	Keitt				Osteen			
Compound	Fresh	50	60	70	Fresh	50	60	70
β-Criptoxanthin	2.18 ± 0.15 ^a^	1.43 ± 0.13 ^b^	0.57 ± 0.14 ^c^	0.85 ± 0.14 ^b^	0.68 ± 0.11 ^a^	0.39 ± 0.03 ^b^	0.20 ± 0.02 ^c^	0.52 ± 0.12 ^a^
13-*cis-*β-Carotene	0.17 ± 0.01 ^a^	0.09 ± 0.05 ^b^	0.03 ± 8.20 ^c^	0.07 ± 0.01 ^b^	0.07 ± 0.11 ^a^	n.d.	n.d.	n.d.
β-Carotene	5.78 ± 0.22 ^a^	3.27 ± 0.43 ^b^	1.73 ± 0.41 ^c^	2.92 ± 0.12 ^b^	3.25 ± 0.42 ^a^	2.22 ± 0.30 ^b^	1.28 ± 0.06 ^c^	3.01 ± 0.51 ^a^
9-*cis*-β-Carotene	1.32 ± 0.06 ^a^	0.64 ± 0.05 ^b^	0.39 ± 0.10 ^c^	0.61 ± 0.05 ^b^	0.48 ± 0.03 ^a^	0.25 ±0.03 ^b^	0.13 ± 0.02 ^c^	0.32 ± 0.06 ^b^
Luteoxanthin	1.33 ± 0.15 ^a^	1.06 ± 0.02 ^a^	0.41 ± 0.05 ^b^	0.65 ± 0.10 ^b^	0.79 ± 0.09 ^a^	0.18 ± 0.02 ^b^	0.08 ± 0.01 ^c^	0.07 ± 0.01 ^d^
Violaxanthin	1.12 ± 0.04 ^a^	0.72 ± 0.08 ^b^	0.36 ± 0.12 ^c^	0.36 ± 0.04 ^c^	0.88 ± 0.08 ^a^	0.12 ± 0.01 ^b^	0.12 ± 0.01 ^b^	n.d.
Neoxanthin	0.38 ± 0.04 ^a^	0.18 ± 0.02 ^b^	0.13 ± 0.02 ^b^	0.16 ± 0.01 ^b^	0.39 ± 0.08 ^a^	n.d.	n.d.	n.d.
Totals	12.28 ± 0.33 ^a^	7.39 ± 0.61 ^b^	3.62 ± 1.66 ^c^	5.62 ± 0.14 ^c^	6.54 ± 0.48 ^a^	3.16 ± 0.32 ^b^	1.81 ± 0.10 ^c^	3.92 ± 0.69 ^b^

Different letters in each cultivar within the same row indicate a significant difference (*p* < 0.05); n.d.: not detected.

**Table 2:** Retinol equivalent (R.E.) (µg/100 g f.w.) and Recommended Daily Allowance (R.D.A.) provided by 100 g and a portion of fresh and dried mangoes.

	Keitt			Osteen				
	Fresh	50	60	70	Fresh	50	60	70
R.E. (µg/100 g f.w.)	215 ± 15	647 ± 70	325 ± 18	537 ± 21	110 ± 13	379 ± 40	211 ± 11	499 ± 60
% R.D.A	27	81	41	67	14	47	26	62
R.E. µg per portion) *	323	194	98	161	165	114	63	150
% R.D.A (per portion)	41	24	12	20	21	14	8	19

R.E.: retinol equivalents; R.D.A: Recommended Daily Allowance (800 μg/die) (Regulation EU No 1169/2011). * 150 g for fresh mango and 30 g for dried mango (LARN (Intake Levels of Reference of Nutrients and energy), 2014).

**Table 3:** Odor threshold, odorant series and odor activity value (OAV) of volatile compounds in fresh and dried (50, 60 and 70 °C) Keitt and Osteen mangoes.

				OAV *							
Compounds	Odorant Series	Odor Threshold (ppb)	Reference	*Keitt*				*Osteen*			
				Fresh	50 °C	60 °C	70 °C	Fresh	50 °C	60 °C	70 °C
Hexadecanoic acid	Berry	8	Hempfling et al. [37]	27.2	0.3	0.3	0.5	10.3	20.3	14.3	13.1
1-Hexanol	Herbaceous, grass, Floral	9	Liu et al. [41]	8.6	0.7	0.5	0.5	0.0	0.4	0.2	0.1
1-Octanol	Jasmine, lemon	190	Pino and Mesa [40]	0.3	0.1	0.0	0.0	17.9	0.0	0.0	0.0
1-Dodecanol	Floral, fruity, fatty	1.5	Yang et al. [36]	14.5	5.1	4.3	1.6	9.8	10.8	2.8	4.3
Hexanal	Fatty, grass, green	4.5	Bonneau et al. [18]	0.0	0.0	0.0	0.7	0.0	0.0	1.5	0.7
Heptanal	Fatty, rancid, citrus Green	3	Bonneau et al. [18]	0.0	6.2	2.9	1.2	0.0	0.0	0.0	2.9
1-Octanal	Honey, green, fruity	0.7	Pino and Mesa. [40]	34.6	0.0	0.0	0.0	68.0	0.0	0.0	3.8
Nonanal	Fatty, wax, citrus, green	1	Bonneau et al. [18]	115.2	208.4	51.8	67.4	159.9	56.0	29.1	35.7
Decanal	Fruity, citrusy, orange	6	Liu et al. [41]	6.7	3.6	1.7	3.1	15.2	2.2	1.7	0.8
trans-2-Nonenal	Fatty, tallowy, metallic	0.08	Pino and Mesa [40]	0.0	71.3	26.3	32.3	0.0	239.9	48.3	23.8
Ethyl butanoate	Apple, fruity	1	Pino and Mesa [40]	0.0	0.0	225.1	93.6	0.0	153.4	0.0	0.0
Ethyl hexanoate	Apple peel, fruity	1	Pino and Mesa [40]	0.0	10.5	41.1	17.2	0.0	35.4	0.0	0.9
Ethyl heptanoate	Brandy, fruity	2	Pino and Mesa [40]	0.0	3.0	2.4	0.7	0.0	0.0	0.0	0.0
Ethyl 3-hydroxy but.	Fruity, grape	1	Moyano et al. [35]	25.4	1.5	5.3	0.0	112.8	0.0	0.0	0.0
γ-Octalattone	Coconut, fatty	7	Pino and Mesa [40]	36.7	2.1	3.7	2.0	0.0	0.1	0.0	0.0
Geranyl acetone	Magnolia, green	60	Pino and Mesa [40]	2.4	0.1	0.1	0.0	0.5	0.1	0.1	0.0
β-Ionone	Dried fruit, woody	0.007	Zhu et al. [38]	11,135.8	552.6	925.4	129.5	2163.9	135.5	44.5	25.8
α-pinene	Pine, grassy	6	Liu et al. [41]	365.3	189.0	904.7	67.1	4335.8	484.7	165.6	32.1
α-fenchene	Camphoraceous, sweet	240	Tamura et al. [39]	1.3	3.8	7.4	0.4	13.4	17.4	0.4	0.2
a-terpinene	Lemony, citrusy	85	Liu et al. [41]	1.9	0.4	2.9	0.1	3.6	2.5	1.5	0.0
D-Limonene	Orange peel-like, citrusy	26	Liu et al. [41]	5.1	1.8	9.2	0.5	43.4	6.1	1.5	0.0
β-Phellandrene	Herbaceous, turpentine	500	Bonneau et al. [18]	0.2	0.1	0.3	0.0	1.0	0.1	0.0	0.0
α-terpinolene	Coniferous odor, rosin-like	140	Liu et al. [41]	1.4	1.0	3.5	0.2	14.0	3.5	0.4	0.0
Isoterpinolene	Citrusy, pine	40	Padrayuttawat et al. [34]	0.4	1.1	0.5	0.4	0.7	1.1	0.1	0.0
Phenol	Phenolic	0.046	Yang et al. [36]	208.0	35.3	27.1	12.4	124.5	23.1	17.1	9.8
β-Caryophyllene	Spicy, woody, green	64	Bonneau et al. [18]	0.0	1.0	0.8	0.2	1.8	7.0	0.5	0.2
α-Caryophyllene	Spicy, woody, oily	160	Bonneau et al. [18]	0.1	0.2	0.2	0.0	10.5	1.3	0.1	0.0

* OAV: odor activity value was calculated by dividing the concentration of an odorant by its orthonasal odor threshold.

## Supplementary Materials

The following are available online at https://www.mdpi.com/2304-8158/9/10/1424/s1, Table S1: Volatile compounds (µg/kg d.b.) in fresh and dried Keitt and Osteen mangoes.
