# Supplementary material for: Evolution of Carotenoid Content, Antioxidant Activity and Volatiles Compounds in Dried Mango Fruits (Mangifera Indica L.)
Source: Foods. 2020 Oct 9;9(10):1424. doi: 10.3390/foods9101424 (PMC7600135; doi:10.3390/foods9101424)
Supplement: Supplementary file 1 [file foods-09-01424-s001.pdf]

Supplementary Table 1. Volatile compounds (μg/Kg d.b.) in fresh and dried *Keitt* and *Osteen* mangoes.

	Keitt				Osteen			
	**Fresh**	**50 °C**	**60 °C**	**70 °C**	**Fresh**	**50 °C**	**60 °C**	**70 °C**
**Acids**								
Acetic acid	n.d. a	1.4 ± 0.03b	n.d. a	1.25 ± 0.03c	25.7 ± 0.6d	2.88 ± 0.07 b	4.7 ± 0.1c	1.38 ± 0.03a
Butanoic acid	32 ± 2c	4.1 ± 0.3a	14.9 ± 1.1b	8.6 ± 0.6a	11.5 ± 0.8c	4.6 ± 0.3b	3.3 ± 0.2ab	1.6 ± 0.1a
Hexanoic acid	51 ± 4b	2.7 ± 0.2a	6.7 ± 0.5a	3.2 ± 0.2a	5.6 ± 0.4c	3.0 ± 0.2b	1.4 ± 0.1a	0.79 ± 0.06a
Heptanoic acid	3.3 ± 0.1d	2.6 ± 0.1c	1.8 ± 0.08b	0.73 ± 0.03a	3.4 ± 0.1c	0.77 ± 0.03 b	0.48 ± 0.02ab	0.24 ± 0.01a
Octanoic acid	25 ± 1c	5.9 ± 0.3b	3.6 ± 0.2ab	1.29 ± 0.07a	7.3 ± 0.4c	1.8 ± 0.1b	1.03 ± 0.06ab	0.67 ± 0.04a
Nonanoic acid	9.2 ± 0.5b	15.8 ± 0.8c	3.3 ± 0.2a	2.3 ± 0.1a	5.8 ± 0.3c	1.54 ± 0.08 b	1.01 ± 0.05ab	0.66 ± 0.04a
Decanoic acid	32 ± 2c	8.7 ± 0.5b	8.3 ± 0.4ab	4.7 ± 0.2a	17.5 ± 0.9c	9.9 ± 0.5b	2.5 ± 0.1ab	3.2 ± 0.2a
Dodecanoic acid	27 ± 1b	n.d. a	n.d. a	n.d. a	7.2 ± 0.3c	9.9 ± 0.4d	0.78 ± 0.03a	5 ± 0.2b
Tetradecanoic acid	94 ± 4b	n.d. a	n.d. a	2.6 ± 0.1a	25.4 ± 1.2b	77 ± 4d	4.0 ± 0.2a	42 ± 2c
Pentadecanoic acid	10.6 ± 0.5b	n.d. a	n.d. a	n.d. a	5.9 ± 0.3c	8.1 ± 0.4d	0.65 ± 0.03a	4.5 ± 0.2b
Hexadecanoic acid	217 ± 9b	2.6 ± 0.1a	2.5 ± 0.1a	4.3 ± 0.2a	173 ± 7b	163 ± 7b	115 ± 5a	105 ± 5a
Heptadecanoic acid	n.d. a	0.8 ± 0.02b	n.d. a	n.d. a	n.d. a	2.86 ± 0.07 d	0.51 ± 0.01b	2.5 ± 0.06c
Octadecanoic acid	48 ± 3b	2.7 ± 0.1a	1.4 ± 0.07a	1.3 ± 0.07a	22.9 ± 1.2b	19.6 ± 1.0b	2.7 ± 0.1a	20 ± 1b
trans-9-Octadecenoic acid	n.d. a	0.8 ± 0.02b	2.3 ± 0.05a	0.9 ± 0.02b	n.d. a	9.0 ± 0.2c	1.28 ± 0.03b	10.4 ± 0.2d
Total	549 ± 27b	48 ± 3a	45 ± 3a	31.4 ± 1.7a	312 ± 14c	314 ± 14c	139 ± 6a	197 ± 9b
	10.53%d	1.51%b	0.90%a	3.16%c	0.84%a	3.27%a	8.44%b	36.02%c
**Alcohols**								
Isoamylalcohol	n.d. a	17 ± 0.6c	n.d. a	2.4 ± 0.09b	n.d. a	1.61 ± 0.06 b	6.1 ± 0.2c	1.5 ± 0.05b
1-Pentanol	n.d. a	1.9 ± 0.07c	n.d. a	1.5 ± 0.06b	n.d. a	n.d. a	n.d. a	0.7 ± 0.03b
cis-Penten-1-ol	47 ± 2b	1.5 ± 0.06a	3.7 ± 0.1a	0.8 ± 0.03a	5.6 ± 0.2d	3.5 ± 0.1c	1.9 ± 0.07b	0.72 ± 0.03a
1-Hexanol	78 ± 3b	6.1 ± 0.3a	4.6 ± 0.2a	4.3 ± 0.2a		3.5 ± 0.2c	1.9 ± 0.08b	1.1 ± 0.05b
1-Octanol	55 ± 2c	18 ± 0.6b	5.7 ± 0.2a	3.9 ± 0.1a	191 ± 11b	4.4 ± 0.1a	4.0 ± 0.1a	2.94 ± 0.09a
1-Nonanol	29.4 ± 0.7b	36 ± 0.9c	5.2 ± 0.1a	4.7 ± 0.1a	20.2 ± 0.5c	2.75 ± 0.07 b	1.2 ± 0.03a	1.09 ± 0.03a
3,6-Nonadien-1-ol	132 ± 7b	4.4 ± 0.2a	2.1 ± 0.1a	0.8 ± 0.04a	35 ± 2b	4.0 ± 0.2a	1.0 ± 0.05a	0.37 ± 0.02a

2,6-Nonadienol	n.d. a	5.9 ± 0.1b	n.d. a	n.d. a	n.d. a	2.47 ± 0.06c	0.51 ± 0.01b	
2-Phenylethylalcohol	n.d. a	1.4 ± 0.03b	2.57 ± 0.06c	n.d. a	n.d. a	0.35 ± 0.01 b	0.4 ± 0.01b	0.24 ± 0.01b
1-Dodecanol	22 ± 1c	7.6 ± 0.4b	6.5 ± 0.3b	2.5 ± 0.1a	14.7 ± 0.7b	16.2 ± 0.8b	4.2 ± 0.2a	6.5 ± 0.3a
Total	362 ± 16c	99 ± 3b	30 ± 1a	20.9 ± 0.8a	266 ± 14c	39 ± 2b	21.2 ± 0.8ab	15.2 ± 0.6a
	6.96%d	3.12%c	0.61%a	2.10%b	0.72%b	0.40%a	1.29%c	2.77%d
**Aldehydes**								
Hexanal	n.d. a	n.d. a	n.d. a	2.9 ± 0.07b	n.d. a	n.d. a	6.6 ± 0.2c	2.93 ± 0.07b
Heptanal	n.d. a	18.7 ± 0.7d	8.8 ± 0.3c	3.7 ± 0.1b	n.d. a	n.d. a	n.d. a	8.8 ± 0.3b
1-Octanal	24.2 ± 0.7b	n.d. a	n.d. a	n.d. a	48 ± 1b	n.d. a	n.d. a	2.64 ± 0.08a
Nonanal	115 ± 5b	208 ± 9c	52 ± 2a	67 ± 3a	160 ± 7c	56 ± 2b	29 ± 1a	36 ± 2a
Furfuraldehyde	47 ± 2c	5.1 ± 0.3b	n.d. a	4.4 ± 0.2ab	7.6 ± 0.4c	n.d. a	n.d. a	2.8 ± 0.2b
Decanal	40 ± 1d	21.7 ± 0.5c	10.4 ± 0.2a	18.5 ± 0.4b	91 ± 2c	13.2 ± 0.3a	10.2 ± 0.2a	4.5 ± 0.1b
Benzaldehyde	21.2 ± 0.8c	10.4 ± 0.4b	18 ± 0.7c	7.5 ± 0.3a	244 ± 9b	3.9 ± 0.2a	3.7 ± 0.1a	1.96 ± 0.08a
trans-2-Nonenal	n.d. a	5.7 ± 0.1d	2.1 ± 0.05b	2.6 ± 0.06c	n.d. a	19.2 ± 0.5d	3.9 ± 0.09c	1.9 ± 0.05b
2,6-Nonadienal	n.d. a	7.0 ± 0.2c	14 ± 0.3d	2.4 ± 0.06b	n.d. a	8.8 ± 0.2d	2.11 ± 0.05c	1.53 ± 0.04b
Total	248 ± 10b	277 ± 11b	105 ± 4a	109 ± 4a	551 ± 20c	101 ± 4b	55 ± 2a	63 ± 2b
	4.75%b	8.71%c	2.13%a	11.02%d	1.49%a	1.05%a	3.37%c	11.45%d
**Aromatic Hydrocarbons**								
2,5-Dimethylstyrene	41 ± 1c	18.2 ± 0.7b	54 ± 2d	3.6 ± 0.1a	193 ± 7c	26 ± 1b	4.0 ± 0.2a	n.d. a
	0.78%c	0.57%b	1.09%d	0.36%a	0.52%b	0.27%a	0.24%a	0%a
**Esters**								
Ethyl butanoate	n.d. a	n.d. a	225 ± 5c	94 ± 2b	n.d. a	153 ± 4b	n.d. a	n.d. a
Ethyl hexanoate	n.d. a	10.5 ± 0.5b	41 ± 2d	17.2 ± 0.8c	n.d. a	35 ± 2b	n.d. a	0.92 ± 0.04a
Ethyl heptanoate	n.d. a	6.1 ± 0.2d	4.8 ± 0.1c	1.37 ± 0.03b	n.d. a	n.d. a	n.d. a	n.d. a
Ethyl octanoate	34 ± 2b	26 ± 1b	83 ± 4c	7.7 ± 0.4b	11.8 ± 0.5b	22 ± 1c	1.45 ± 0.07a	n.d. a
cis-3-Hexenyl Butyrate	61 ± 2c	4.7 ± 0.2a	23.6 ± 0.9b	n.d. a	n.d. a	n.d. a	n.d. a	n.d. a
Ethyl 3-hydroxy butanoate	25 ± 1c	1.52 ± 0.08a	5.3 ± 0.3b	n.d. a	113 ± 6b	n.d. a	n.d. a	n.d. a
-Butyrolactone	9.3 ± 0.4a	13.0 ± 0.5b	17.3 ± 0.7c	11.8 ± 0.5b	4.7 ± 0.2c	8.8 ± 0.3b	26 ± 1c	7.1 ± 0.3b
Ethyl decanoate	17 ± 1c	4.5 ± 0.3a	8.2 ± 0.6b	3.3 ± 0.2a	n.d. a	5.4 ± 0.4c	0.9 ± 0.06b	0.67 ± 0.05b
Ethyl benzoate	3.2 ± 0.2c	1.12 ± 0.06b	3.3 ± 0.2c	n.d. a	18 ± 1b	1.65 ± 0.08a	n.d. a	n.d. a
Diethyl succinate	n.d. a	0.8 ± 0.02b	5.2 ± 0.1d	2.28 ± 0.05c	n.d. a	3.69 ± 0.09c	n.d. a	0.78 ± 0.02b
Ethyl dodecanoate	12.7 ± 0.6b	4.7 ± 0.2a	19.9 ± 0.9c	4.9 ± 0.2a	n.d. a	4.0 ± 0.2c	0.52 ± 0.02b	0.46 ± 0.02b
γ-Octalattone	257 ± 11b	14.7 ± 0.7a	26 ± 1a	13.9 ± 0.6a	n.d. a	0.69 ± 0.03c	0.19 ± 0.01b	0.16 ± 0.01b
Ethyl tetradecanoate	22 ± 1ab	22 ± 1a	101 ± 4c	32 ± 1b	3.9 ± 0.2b	19.0 ± 0.8d	6.9 ± 0.3c	1.9 ± 0.08a
Ethyl pentadecanoate	n.d. a	0.57 ± 0.01b	n.d. a	n.d. a	n.d. a	0.6 ± 0.01c	0.16 ± 0.01b	0.10 ± 0.01b
Ethyl hexadecanoate	10.1 ± 0.6c	6.4 ± 0.4b	11.2 ± 0.7c	3.7 ± 0.2a	8.7 ± 0.5b	9.9 ± 0.6b	3 ± 0.2a	2.0 ± 0.1a
Ethyl octadecanoate	n.d. a	n.d. a	n.d. a	n.d. a	n.d. a	1.0 ± 0.02c	0.51 ± 0.01b	0.25 ± 0.01b

Total	452 ± 21c	117 ± 5a	575 ± 21d	192 ± 7b	159 ± 8c	266 ± 9d	39 ± 2b	14.3 ± 0.6a
	8.68%b	3.68%a	11.66%c	19.31%d	0.43%a	2.76%c	2.39%c	2.62%c
**Ketones**								
Geranyl acetone	142 ± 5b	4.6 ± 0.2a	3.50 ± 0.13a	1.88 ± 0.07a	32 ± 1c	8.1 ± 1.2b	6.4 ± 0.2b	0.88 ± 0.03a
β-Ionone	78 ± 3b	3.9 ± 0.1a	6.5 ± 0.2a	0.91 ± 0.03a	15.2 ± 0.6b	0.9 ± 0.6a	0.31 ± 0.01a	0.18 ± 0.01a
Total	220 ± 8b	8.5 ± 0.3a	10.0 ± 0.4a	2.8 ± 0.1a	48 ± 2c	9.1 ± 1.7b	6.7 ± 0.2b	1.06 ± 0.04a
	4.23% c	0.27%b	0.20%a	0.28%b	0.13%b	0.09%a	0.41%d	0.19%c
**Monoterpenes**								
α-pinene	2192 ± 67d	1134 ± 35b	1340 ± 41c	403 ± 12a	26015 ± 800b	2908 ± 89b	994 ± 31a	193 ± 6a
α-fenchene	311 ± 12d	921 ± 35c	1772 ± 68d	92 ± 4a	3207 ± 122b	4165 ± 159c	90 ± 3a	46.9 ± 1.8a
α-terpinene	163 ± 4d	30 ± 0.7b	46 ± 1c	9.0 ± 0.2a	310 ± 7d	209 ± 5c	131 ± 3b	n.d. a
D-Limonene	132 ± 6d	46 ± 2c	33 ± 1b	12.0 ± 0.5a	1128 ± 50c	158 ± 7b	38 ± 2a	n.d. a
β-Phellandrene	95 ± 4c	66 ± 3b	127 ± 5d	22 ± 1a	524 ± 23b	43 ± 2a	8.5 ± 0.4a	n.d. a
γ-Terpinene	188 ± 5c	7.7 ± 0.2a	26.8 ± 0.6b	4.6 ± 0.1a	33.7 ± 0.8c	23.5 ± 0.6b	1.13 ± 0.03a	n.d. a
δ-3-carene	n.d. a	3.6 ± 0.2a	56 ± 2b	1.32 ± 0.06a	n.d. a	18.9 ± 0.8b	0.88 ± 0.04a	n.d. a
β-ocimene	n.d. a	19.5 ± 0.9b	55 ± 3c	4.8 ± 0.2a	n.d. a	25 ± 1c	2.9 ± 0.1b	0.18 ± 0.01a
α-terpinolen	199 ± 5c	141 ± 3b	494 ± 12d	28.4 ± 0.7a	1959 ± 47c	492 ± 12b	58.6 ± 1.4a	0.86 ± 0.02
Isoterpinolene	14.1 ± 0.5a	46 ± 2c	19.1 ± 0.7ab	17.6 ± 0.7b	29 ± 1c	45 ± 2d	5.3 ± 0.2b	
Total	3294 ± 103c	2416 ± 82b	3969 ± 135d	595 ± 19a	33205 ± 251c	8088 ± 278b	1330 ± 41b	241 ± 8a
	63.24 %b	75.96%c	80.44%d	59.94%a	89.57%c	84.19%b	80.80%b	43.90%a
**Phenols**								
Phenol	9.57 ± 0.66c	1.6 ± 0.1a	1.25 ± 0.09a	0.57 ± 0.04a	5.7 ± 0.4b	1.06 ± 0.07a	0.79 ± 0.05a	0.45 ± 0.03a
	0.18% b	0.05%a	0.03%a	0.06%a	0.02%a	0.01%a	0.05%b	0.08%b
**Sesquiterpenes**								
α-Copaene	n.d. a	45 ± 1d	26.1 ± 0.6c	8.1 ± 0.2b	n.d. a	2.41 ± 0.06 b	n.d. a	n.d. a
α-Gurjunene	12.3 ± 0.5c	8.82 ± 0.39b	9.15 ± 0.40b	n.d. a	30.07 ± 1.33c	14.02 ± 0.62 b	2.3 ± 0.1a	n.d. a
β-Caryophyllen	n.d. a	64 ± 1d	51 ± 1c	13.7 ± 0.3b	113 ± 2.7b	450 ± 11c	30 ± 0.7c	15.9 ± 0.4c
Humulen	n.d. a	3.45 ± 0.08d	4.1 ± 0.1c	1.58 ± 0.04b	80 ± 2c	11.4 ± 0.3b	1.87 ± 0.04a	n.d. a
α-Caryophyllene	9.1 ± 0.2b	33 ± 0.8d	24.6 ± 0.6c	6.9 ± 0.2a	1675 ± 40c	212 ± 5b	13.4 ± 0.3a	0.30 ± 0.01a
β-Selinene	11.8 ± 0.5b	33 ± 1d	25 ± 1c	5.4 ± 0.2a	325 ± 14c	63 ± 3b	2.02 ± 0.09a	n.d. a
Valencene	n.d. a	3.05 ± 0.07d	2.76 ± 0.07c	1.08 ± 0.03b	33.8 ± 0.8c	3.82 ± 0.09 b	n.d. a	n.d. a
α-Selinene	n.d. a	4.4 ± 0.1d	1.97 ± 0.05c	0.60 ± 0.01b	40 ± 1c	5.7 ± 0.1b	n.d. a	n.d. a
Eremophilene	n.d. a	n.d. a	n.d. a	n.d. a	32.3 ± 0.8b	1.07 ± 0.03a	n.d. a	n.d. a
Total	33 ± 1a	195 ± 5c	145 ± 4b	37 ± 1a	2330 ± 63c	764 ± 20b	50 ± 1a	16.2 ± 0.4a
	0.64%a	6.14%d	2.93%b	3.76%c	6.28%b	7.95%c	3.02%a	2.96%a

Total Vocs	5209c	3181b	4934c	993a	37069d	9607c	1647b	548a

Significance, analysis of variance (ANOVA) with the honestly significant difference (HSD) in a Tukey test at different significance levels, between different drying treatments and fresh fruit ($p < 0.05$)
n.d.: not determinable
